# Low-Grade Uterine Epithelioid Hemangioendothelioma Presented as a Submucosal Leiomyoma during Labor

**DOI:** 10.1155/2013/423584

**Published:** 2013-01-20

**Authors:** Anastasios V. Koutsopoulos, Efthimios Sivridis, Panagiotis Tsikouras, Vasileios Liberis, Georgia Karpathiou, Alexandra Giatromanolaki

**Affiliations:** ^1^Department of Pathology, Faculty of Medicine, Democritus University of Thrace and University General Hospital of Alexandroupolis, Thrace, 68100 Alexandroupolis, Greece; ^2^Department of Obstetrics and Gynecology, Faculty of Medicine, Democritus University of Thrace and University General Hospital of Alexandroupolis, Thrace, 68100 Alexandroupolis, Greece

## Abstract

With the exception of leiomyomas, soft tissue tumors of the uterine corpus are not common. This is particularly true for vascular neoplasms, with the epithelioid hemangioendothelioma being a curiosity; not more than twenty-two cases of malignant hemangioendotheliomas have been reported in the literature so far, all of which were high-grade hemangioendotheliomas (hemangiosarcomas). We present herewith a unique case of low-grade epithelioid hemangioendothelioma of the uterus in a pregnant woman aged 29 years. The clinical, histological, and immunohistochemical characteristics of this entity, together with its differential diagnosis, are discussed.

## 1. Introduction

Leiomyomas are common uterine neoplasms, but other, benign or malignant, soft tissue tumors are very rare [1]. Epithelioid hemangioendotheliomas are even more rare [2], with only twenty-two cases having been published in the English medical literature, and these were all malignant [3–5].

We present herewith a case of uterine epithelioid hemangioendothelioma of low-grade malignancy, the first reported in the English language literature, hoping that it will contribute to the general knowledge within the spectrum of epithelial vascular neoplasms.

## 2. Case Presentation

A twenty-nine-year-old pregnant woman, at 32-month of gestation, was admitted to the Department of Obstetrics and Gynecology, because of premature rupture of fetal membranes, which led to urgent cesarean delivery. Physical examination did not reveal other pathological findings. Patient's personal history was unremarkable, having no previous surgery or use of hormonal drugs. The patient was nulliparous and had no abortions. During surgery, a submucosal endometrial mass was detected in the region of isthmus, which was taken as a leiomyoma. Excision of the tumor was performed and was sent to the Department of Pathology for histopathologic evaluation.

## 3. Pathologic Examination

Macroscopic examination of the excised specimen showed a relatively circumscribed tumor, 1.5 cm in diameter, of soft consistency. Formalin-fixed, paraffin-embedded tissue sections were stained with hematoxylin and eosin and assessed immunohistochemically for the following markers: CD31 (DAKO, clone JC70A, dilution 1 : 20), Vimentin (DAKO, clone V9, dilution 1 : 100), CD34 (NOVOCASTRA, clone QBEnd/10, dilution 1 : 50), SMA (NOVOCASTRA, clone asm-1, dilution 1 : 50), Myosin (DAKO, clone SMMS-1, dilution 1 : 70), Desmin (NOVOCASTRA, clone DE-R-11, dilution 1 : 70), Cytokeratin (DAKO, clone MNF116, dilution 1 : 70), CD10 (DAKO, clone 56C6, dilution 1 : 100), EMA (NOVOCASTRA, clone GP1.4, dilution 1 : 300), S-100 protein (NOVOCASTRA, polyclonal, dilution 1 : 300), and Ki-67 (DAKO, clone MIB-1, dilution 1 : 100).

**Figure 1:**
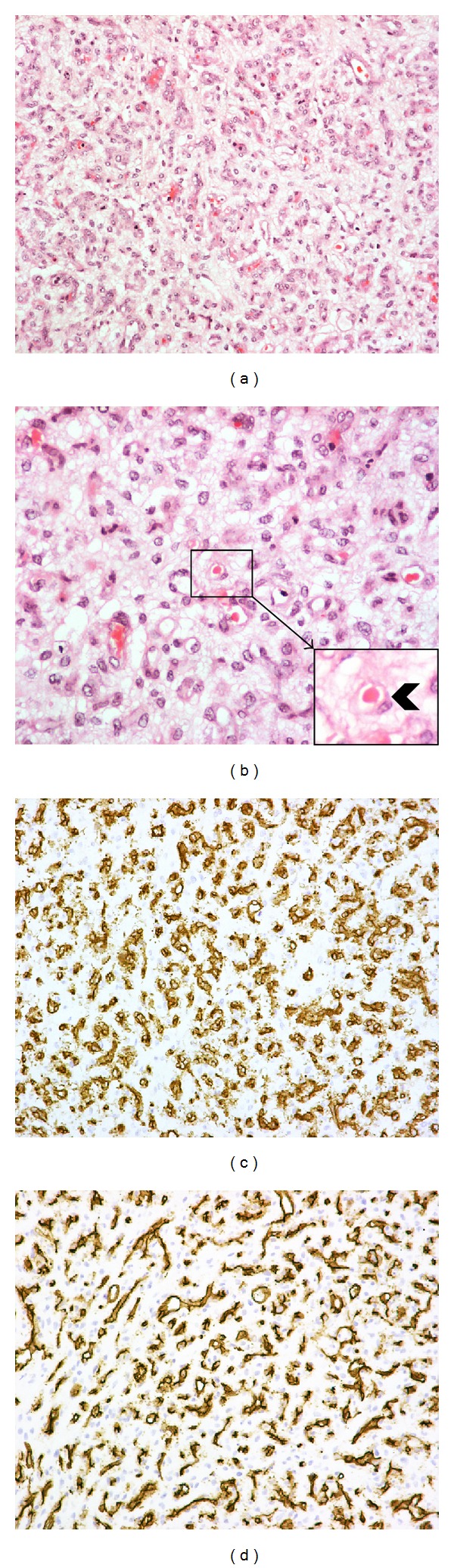
(a) and (b) tissue sections showing well-formed vascular channels embedded in a myxoid stroma. Note focal areas of abortive endothelial differentiation (hematoxylin and eosin stained sections, (a) magnification ×200 and (b) magnification ×400). Insert in (b) displays the presence of an intracytoplasmic vacuole containing a single erythrocyte (arrowhead) (magnification ×600). (c) and (d) neoplastic cells demonstrating strong diffuse immunoreactivity for the endothelial markers CD31 (c) and CD34 (d), magnification ×200.

On microscopic examination, the tumor was composed almost exclusively of well-formed vascular channels embedded in a myxoid stroma (Figure 1(a)). There were, however, a few focal areas with abortive endothelial differentiation (Figures 1(a) and 1(b)). The neoplastic cells were, for the most part, of epitheliod type showing vesicular nuclei, with prominent nucleoli, but little pleomorphism, and a variable amount of eosinophilic cytoplasm. A characteristic feature of the tumor cells was the presence of intracytoplasmic vacuoles containing red blood cells (Figure 1(b) and insert). Mitoses were <5 per ten (10) high power field (×400). Necrosis was not seen. Immunohistochemical analysis showed strong diffuse immunoreactivity for the endothelial markers CD31 (Figure 1(c)), Factor VIII and CD34 (Figure 1(d)), whereas the markers SMA, Myosin, Desmin, MNF116, EMA, CD10, and S-100 protein were negative. The proliferation cell marker MIB-1 was 4%. Reticulin staining demonstrated vasoformative architecture. Most of the tumor surface was covered by decidualized endometrium. The surgical margins were free of tumor.

Clinical and laboratory investigation for extrauterine spread or evidence of distant metastases was negative. No further therapy was administered. The patient is free of disease ten months postoperatively.

## 4. Discussion

Epithelioid hemangioendotheliomas are soft tissue tumors of uncertain biological behavior; they are generally considered as being borderline tumors lying within the spectrum of vascular tumors, at one end of which are hemangiomas and at the other end are high-grade epithelioid hemangioendotheliomas, also called hemangiosarcomas [6, 7]. Such tumors, not only occur in uterine tissues, but are also seen in organs such as lung, liver, bone, pleura, peritoneum, skin, thyroid gland, lymph node, stomach, brain, and meninges [8, 9]. Up to date, only twenty-two cases of pure uterine hemangioendotheliomas have been reported in the English literature, none of which were low grade. There were patients between the ages of 7–76 years [2, 3, 5, 10, 11]. We present a unique case of a low-grade epithelioid hemangioendothelioma of the uterus in a pregnant woman aged 29 years. The histologic picture was typical of a low-grade epithelioid hemangioendothelioma with numerous well-formed vascular channels having a single erythrocyte within a characteristic cytoplasmic vacuole [7]. There were neither necrosis nor infiltrative borders. The neoplastic endothelial cells showed bland histologic features and a mitotic count <5 per ten (10) high power field (×400). The diagnosis was confirmed by immunohistochemistry.

Although epithelioid hemangioendotheliomas are usually presented with vaginal bleeding, anaemia, weight loss, and enlargement of the uterus [1–3, 11], apparently none of the above characteristics were observed in our case.

The differential diagnosis should always include capillary and epithelioid hemangiomas, primary or metastatic high-grade hemangioendothelioma (angiosarcoma), carcinosarcoma (malignant mixed Mullerian tumor), vascular leiomyosarcoma, and hemangiopericytoma [2, 10]. In contrast to high-grade hemangioendothelioma, the low-grade disease shows little cellular pleomorphism, nuclear hyperchromatism, or necrosis and exhibits fewer solid areas with lower mitotic rate. Immunoreactivity for vascular markers [1, 2] and the absence of stain for epithelial and muscle markers should exclude the diagnosis of carcinosarcoma and vascular leiomyosarcoma. The presence of intracytoplasmic vacuoles in the form of minute lumens with erythrocytes favors endothelial cell differentiation. Electron microscopy may also contribute to the diagnosis if Weibel-Palade bodies can be verified [12–15].

The pathogenesis of the tumor is obscure, although Morrel et al. [12] presented a case of a 61-year-old woman who developed a uterine angiosarcoma 6.5 years following radiotherapy for a squamous cell carcinoma of the uterine cervix. Others incriminated chemotherapy, chronic lymphedema, chronic steroid use, chemical exposure and tumor collision or tumor transformation as being responsible for the tumor development [1, 2, 14, 16]. Yet, our patient had no history of uterine leiomyoma or radiation therapy.

The management of the uterine epithelioid hemangioendothelioma remains under consideration, mainly because of its rarity [1]. Olawaiye et al. [3] suggested that surgical resection of the tumor should be followed by sequential individualized chemotherapy and radiotherapy for high-grade tumors. Antiangiogenic agents may have a merit. The prognosis of epithelioid hemangioendothelioma of high-grade is very poor [10]. Given the indolent course of low-grade epithelioid hemangioendothelioma and the complete excision offered, no further surgical therapy or adjuvant treatment was administered in our case.
